# Natural History of Clinical, Laboratory, and Echocardiographic Parameters of a Primary Hyperoxaluria Cohort on Long Term Hemodialysis

**DOI:** 10.3389/fmed.2021.592357

**Published:** 2021-04-09

**Authors:** David J. Sas, Felicity T. Enders, Tina M. Gunderson, Ramila A. Mehta, Julie B. Olson, Barbara M. Seide, Carly J. Banks, Bastian Dehmel, Patricia A. Pellikka, John C. Lieske, Dawn S. Milliner

**Affiliations:** ^1^Division of Pediatric Nephrology and Hypertension, Mayo Clinic, Rochester, MN, United States; ^2^Department of Laboratory Medicine and Pathology, Mayo Clinic, Rochester, MN, United States; ^3^Division of Nephrology and Hypertension, Mayo Clinic, Rochester, MN, United States; ^4^Division of Biomedical Statistics and Informatics, Mayo Clinic, Rochester, MN, United States; ^5^OxThera AB, Stockholm, Sweden; ^6^Department of Cardiovascular Medicine, Mayo Clinic, Rochester, MN, United States

**Keywords:** primary hyperoxaluria, oxalosis, echocardiography, dialysis, renal replacement therapy 2

## Abstract

**Background:** Primary hyperoxaluria type 1 (PH1) is a rare monogenic disorder characterized by excessive hepatic production of oxalate leading to recurrent nephrolithiasis, nephrocalcinosis, and progressive kidney damage, often requiring renal replacement therapy (RRT). Though systemic oxalate deposition is well-known, the natural history of PH1 during RRT has not been systematically described. In this study, we describe the clinical, laboratory, and echocardiographic features of a cohort of PH1 patients on RRT.

**Methods:** Patients with PH1 enrolled in the Rare Kidney Stone Consortium PH Registry who progressed to require RRT, had ≥2 plasma oxalate (pOx) measurements 3–36 months after start of RRT, and at least one pair of pOx measurements between 6 and 18 months apart were retrospectively analyzed. Clinical, echocardiographic, and laboratory results were obtained from the Registry.

**Results:** The 17 PH1 patients in our cohort had a mean total HD hours/week of 17.4 (SD 7.9; range 7.5–36) and a range of age of RRT start of 0.2–75.9 years. The average change in plasma oxalate (pOx) over time on RRT was −0.74 [−2.9, 1.4] μmol/L/month with the mean pOx never declining below 50 μmol/L. Over time on RRT, oxalosis progressively developed in multiple organ systems. Echocardiography performed on 13 subjects showed worsening of left ventricular global longitudinal strain correlated with pOx (*p* < 0.05).

**Conclusions:** Even when a cohort of PH1 patients were treated with intensified RRT, their predialysis pOx remained above target and they developed increasing evidence of oxalosis. Echocardiographic data suggest that cardiac dysfunction could be related to elevated pOx and may worsen over time.

## Introduction

The primary hyperoxalurias are a group of genetic diseases that result in excessive hepatic oxalate production producing increased urinary oxalate excretion, which can cause severe urinary stone disease, nephrocalcinosis, and progressive chronic kidney disease (CKD). Systemic oxalosis can occur with end-stage kidney disease (ESKD) if patients are maintained on routine renal replacement therapy (RRT) long term. The 3 known genetic causes of primary hyperoxaluria (PH) are caused by mutations in *AGXT* (PH1), *GRHPR* (PH2), and *HOGA1* (PH3).

On average, PH1 manifests the most severe hyperoxaluria and the greatest risk of ESKD. In the cohort of PH1 patients enrolled in the Rare Kidney Stone Consortium (RKSC) registry, 57% progressed to ESKD by 40 years of age and 88% by age 60 (1, 2). Since the vast majority of oxalate is eliminated by the kidney, once patients progress to ESKD plasma oxalate (pOx) concentrations dramatically increase. Once pOx exceeds a critical threshold (believed to be 30–45 μmol/L) (3, 4), systemic oxalosis can occur due to deposition of calcium oxalate crystals in a variety of tissues, including the heart, bone, eyes, skin, blood vessels, endocrine, and nervous systems (2).

Little is known about the natural history of PH1 patients maintained on RRT for a prolonged period of time. Thus, in this study, we characterized the progression of clinical, laboratory, and echocardiographic features of a cohort of PH1 patients maintained on RRT to better understand the expected clinical course including changes in pOx, oxalosis findings, and echocardiogram findings over time.

## Materials and Methods

### Study Population

This was a retrospective observational study. Clinical, laboratory, and echocardiographic information were abstracted from PH1 patients enrolled in the RKSC PH registry between 2003 and 2018 (5) and augmented by review of the medical records as necessary. Informed consent for registry participation was obtained from each subject after Mayo Clinic Institutional Review Board approval. All patients in the current study had confirmed mutations of the *AGXT* gene. We anticipated that pOx would increase over time on RRT as any remaining endogenous kidney function was lost and oligoanuria ensued.

### Renal Replacement Therapy

**Figure 1:**
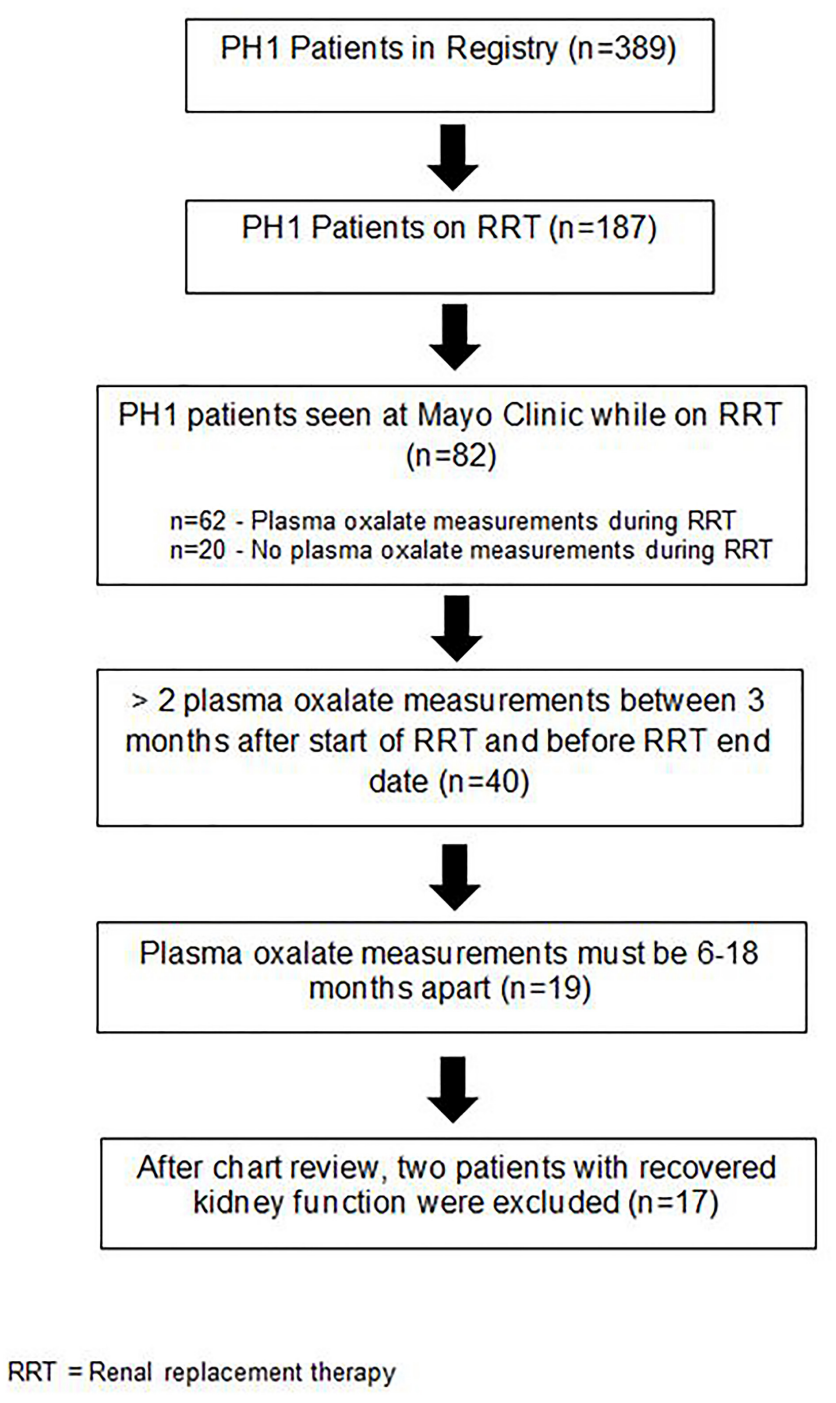
Schematic of subject flow.

A major objective of this study was to define the natural history of pOx over time on RRT. Therefore, in order for to be included in this study, PH1 patients were required to have at least 2 pOx measurements by Mayo Clinic Renal Testing Laboratory between 3 and 36 months after initiation of RRT, with at least one pair of measurements between 6 and 18 months apart (Figure 1). One patient was homozygous for the G170R mutation known to confer responsiveness to this pyridoxine and was receiving pyridoxine at the time of pOx measurements. The 82 PH patients seen at Mayo Clinic on RRT represent heterogenous scenarios which accounts for the decreased sample size meeting our inclusion criteria. For example, some of these patients came to Mayo for a brief period for second opinions or follow-up rather than longer-term care.

Descriptive statistics are provided as counts, mean/standard deviation (SD) and/or median/interquartile range (IQR), and range for continuous variables or counts and percentages for categorical variables. All pOx samples were obtained immediately prior to RRT sessions and measurements were made in a single reference laboratory, Mayo Clinic Renal Testing Laboratory (normal <1.6 μmol/L) (6). Plasma and urine oxalate was measured using an enzymatic oxalate oxidase method (Trinity Biotech, Wicklow, Ireland), as previously described for plasma (6) and urine (7). The test is based upon oxalate reduction by oxalate oxidase yielding hydrogen peroxide, which in the presence of peroxidase reacts with an indamine dye. This colored end point is measured using a sensitive Beckman Coulter DU800 Spectrophotometer at 590 nm.

Of note, though kidney transplant is considered a form of RRT, post-transplant patients were not included in this study. “Standard” RRT regimen is considered 12 h per week, given a typical prescription of 3 weekly sessions, 4 h each, while weekly durations >12 h are considered “intensified.”

### Investigations for Tissue-Specific Oxalosis

Since few patients had undergone biopsies to definitively document the presence of oxalate crystals in extrarenal body tissues clinical findings associated with oxalosis were abstracted as a surrogate. Table 1 details investigations used to evaluate for evidence of systemic oxalosis in specific tissues. Evaluation of bone density by dual energy X-ray absorptiometry (DEXA), cardiac function by electrocardiography (ECG) and echocardiography, and presence of retinal oxalate by funduscopic examination were obtained for clinical indications or as screening for systemic oxalosis. Medical records were reviewed for clinical indicators of systemic oxalate deposition including pathologic fractures, erythropoiesis-stimulating agents (ESA) refractory anemia (defined as significant and persistent anemia despite management with erythropoietin stimulating agents, requiring periodic transfusions), cardiac arrhythmias or cardiomyopathy, livedo reticularis, non-healing ulcers, subcutaneous nodules resembling oxalate deposits, peripheral neuropathy, and persistent musculoskeletal pain and weakness. Bone manifestations for this analysis were limited to pathologic fractures, radiographic evidence suggesting oxalate osteopathy, or bone biopsy showing oxalate induced changes in osteoclasts or trabecular architecture. The majority of patients had these tests performed on a repeated basis while on RRT, although they were not done on a protocolized schedule.

**Table 1:** Investigations used to evaluate for oxalate deposition in specific tissues.

**Tissue**	**Investigation**
Bone	DEXA scan, radiographs, evidence of pathologic fractures, bone, or bone marrow biopsy
Cardiac	ECG, chest radiographs, echocardiography, persistent hypotension
Musculoskeletal	Persistent musculoskeletal pain and weakness
Neurological	Exam or nerve conduction confirming neuropathy
Retina	Funduscopic examination for crystals by ophthalmologist
Skin	Livedo reticularis, subcutaneous nodules resembling oxalate deposits, non-healing ulcers

*DEXA, dual energy x-ray absorptiometry; ECG, electrocardiogram*.

### Echocardiography

Of the 17 subjects, 13 had at least one echocardiogram during follow-up on RRT. All echocardiograms were reviewed by a Mayo Clinic cardiologist (PP). For the purposes of demographics and analysis, a subject's first echocardiogram was designated as the echocardiogram after the start of the RRT. Descriptive statistics are provided as counts, median/IQR, and range. Data were treated as independent points for analysis. Association of echocardiographic indices with duration of RRT was examined using linear regression models.

Windows were treated as 60 day rolling periods. If multiple echocardiograms were performed within the time window, the first was included, as follow up studies were frequently abbreviated.

Indices abstracted for the echocardiogram analysis included left ventricular and right ventricular global longitudinal peak systolic strain (LV global strain, RV global strain), left ventricular ejection fraction (LVEF), left ventricular stroke volume index (LVSVI), left ventricular mass index (LVMI), left atrial volume index (LAVI), the ratio of the mitral inflow early diastolic velocity /medial mitral annulus early diastolic velocity (E/e', an indicator of left ventricular filing pressure), right ventricular systolic pressure (RVSP), and relative apical strain. Because of the young age of our 2 pediatric subjects who had echocardiograms and the age-related differences in echocardiographic measurements, this analysis was only performed on data from adult subjects. Relative apical strain was calculated as:

$$\begin{array}{l} (mean\;of\;apical\;LV\;strain\;segments)/[(mean\;of\;six\;basal\;LV\;strain\\ \;segments)\;+\;(mean\;of\;six\;mid\;LV\;strain\;segments)]. \end{array}$$

## Results

Patient demographics are shown in Table 2. Nineteen subjects with PH1 requiring RRT initially met criteria for inclusion in the analysis, of whom two patients recovered kidney function during RRT. Since this improved kidney function likely altered oxalate dynamics and risk for systemic deposition, these subjects were excluded from the cohort.

**Table 2:** Baseline demographic characteristics of PH1 patients at RRT start date.

	***N* = 17**
**Age at PH1 diagnosis (years)**	
Mean (SD)	22.4 (21.9)
Median (IQR)	18.9 (4.1, 28.9)
Range	0.3–74.0
**Age at RRT start date (years)**	
Mean (SD)	37.4 (24.3)
Median (IQR)	27.4 (21.2, 61.5)
Range	0.2–75.9
**Gender**	
Female	11 (64.7%)
Male	6 (35.3%)

The majority (88.2%) of the cohort was white; 58.8% were white non-Hispanic or Latino, while ethnicity was unknown or not reported in 41.2%. The median age (range) for diagnosis was 19 (0.3–74) years and start of RRT was 27 (0.2–75.9) years. Eleven of the 17 members of our cohort had a diagnosis of PH prior to initiation of RRT; 4 were first known to have PH within 3 months before or after starting RRT (including both infants who first presented in ESKD), and there were 2 in whom the PH diagnosis was made >3 months after the start of dialysis.

Baseline characteristics, RRT regimen, and clinical parameters over time on RRT are shown in Table 3. At baseline, 16 subjects received HD only and one subject received PD only. The mean (SD) days of treatment per week was 4.3 (1.3) (range 3–6 days) and session lengths averaged 3.8 (0.8) h (range 2.5–6.0). Total HD was 17.4 (7.9) h/week (range 7.5–36). Over the course of dialysis, three patients received PD either alone or in conjunction with HD; the remainder received HD only. The decline in sample size each year represents patients discontinuing RRT due to transplant. By 2 years after initiation of dialysis, 11 subjects had been transplanted. A single subject was transplanted between 2 and 3 years and 5 subjects continued on RRT beyond 3 years.

**Table 3:** PH1 patients' RRT regimen, plasma oxalate concentration, and urine output.

	**Baseline (*N* = 17)**	**1 year (*N* = 12)**	**2 years (*N* = 6)**	**3+ years (*N* = 5)**
**Type of RRT**				
HD + PD	0 (0.0%)	2 (16.7%)	0 (0.0%)	0 (0.0%)
HD only	16 (94.1%)	10 (83.3%)	6 (100.0%)	5 (100.0%)
PD only	1 (5.9%)	0 (0.0%)	0 (0.0%)	0 (0.0%)
**RRT treatments/week**				
*N*	15	11	6	5
Median (IQR)	4.0 (3.0, 5.5)	6.0 (4.0, 6.0)	5.0 (3.5, 5.8)	5.0 (3.0, 5.0)
Range	3.0–6.0	3.0–6.0	2.0–6.0	2.0–6.0
**Hours/RRT session**				
*N*	13	11	6	5
Median (IQR)	4.0 (3.5, 4.0)	3.5 (3.0, 4.0)	3.0 (3.0. 3.4)	3.0 (3.0, 3.5)
Range	2.5–6.0	2.5–4.0	3.0–4.0	3.0–4.0
**Hours RRT/week**				
*N*	13	11	6	5
Median (IQR)	18.0 (11.2, 20.0)	18.0 (13.5, 24.0)	15.0 (11.6, 17.2)	15.0 (10.5, 15.0)
Range	7.5–36.0	7.5–24.0	6.0–24.0	6.0–24.0
First pOx measured after starting RRT (μmol/L)^*^				
*N*	17	12	6	5
Median (IQR)	75.0 (40.3, 150.7)	76.0 (37.0, 145.7)	80.1 (44.9, 133.8)	77.0 (34.2, 83.2)
Range	16.1–187.5	16.1–159.8	16.1–159.8	16.1–150.7
Urine (mL/24 h)^**^				
*N*	6	7	3	1
Median (IQR)	3,582 (2,380, 4,243)	1,300 (798, 1,848)	2,250 (1,204, 2,409)	1,658 (1,658, 1,658)
Range	28.0–4,637	384–2,392	157–2,568	1,658–1,658

* *pOx reference range <1.6 μmol/L*.

** *A single data point in this set is from a pediatric patient and this volume was not corrected for BSA*.

**Figure 2:**
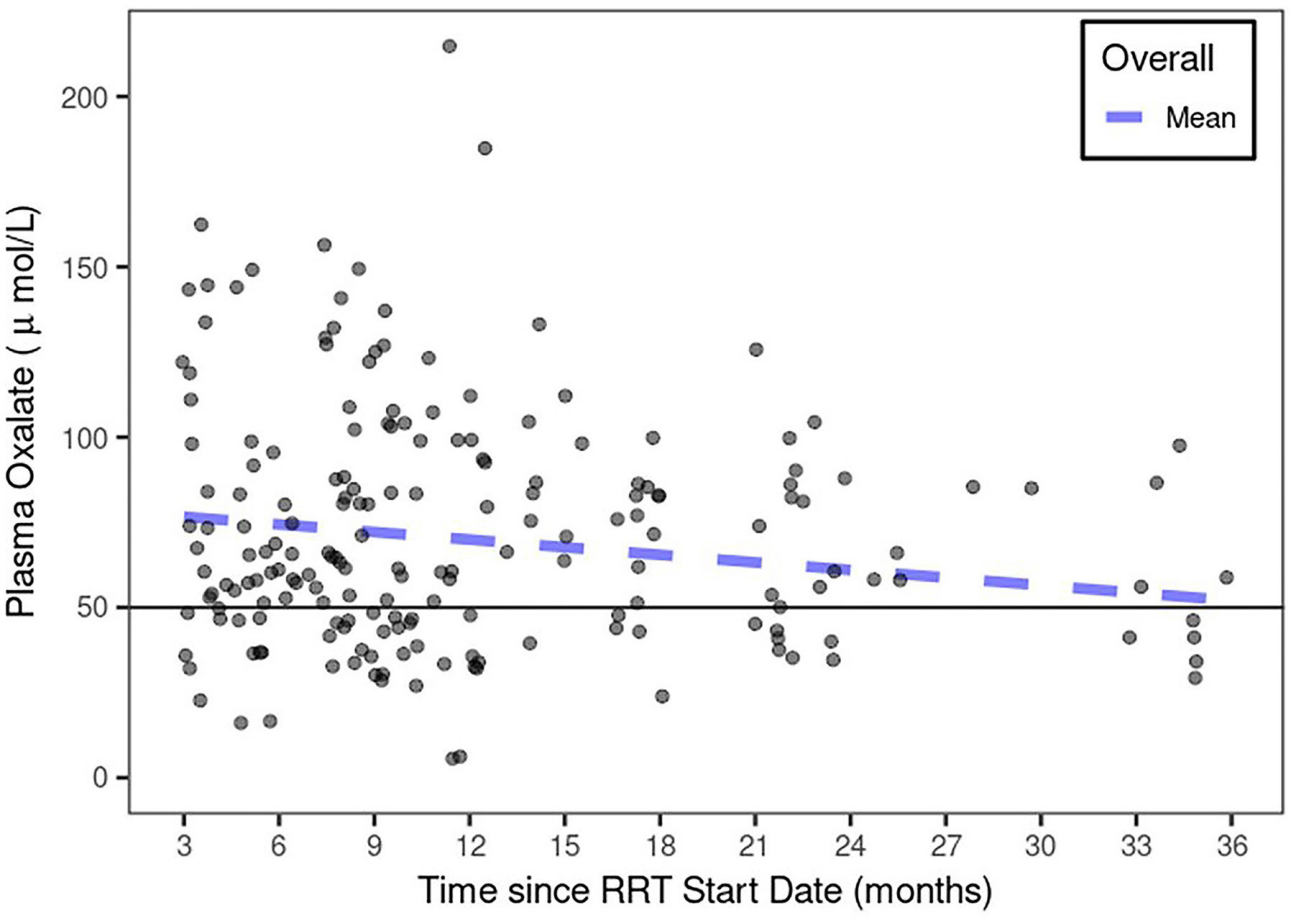
pOx over the course of RRT in PH1. The mean pOx declined slightly over time (though not statistically significant) with RRT, but remained above 50 μmol/L using a linear mixed-effects model with subject-specific intercept and slopes.

**Figure 3:**
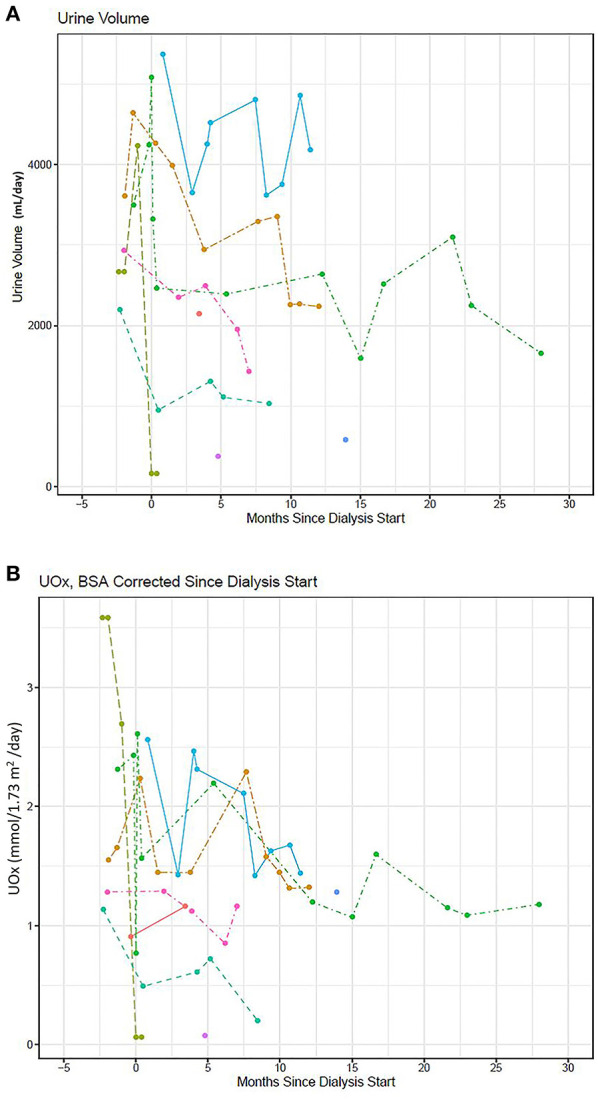
**(A)** Urine output (mL/day) and **(B)** urine oxalate excretion/BSA (mmol/1.73m^2^/day) over time since initiating RRT (*n* = 9).

The average change in pOx over the course of RRT was −0.74 [−2.9, 1.4] μmol/L/month with the mean pOx never decreasing below 50 μmol/L despite RRT regimens with greater weekly duration than are standard (Figure 2). Median pOx was slightly lower immediately after initiation of RRT compared to the first measurement included in the analysis (i.e., >90 days after start of RRT) (75.0–80.3 μmol/L). Urine output was maintained in 6/17 subjects, 3 of whom still had >2000 ml/day after 1 year on dialysis (Figure 3A). Oxalate excretion ranged from 0.85 to 2.3 mmol/1.73 m^2^/day measured in 5 subjects 6 months after the start of dialysis and 1.2–1.4 mmol/1.73m^2^/day at 1 year (*n* = 3) (Figure 3B).

**Figure 4:**
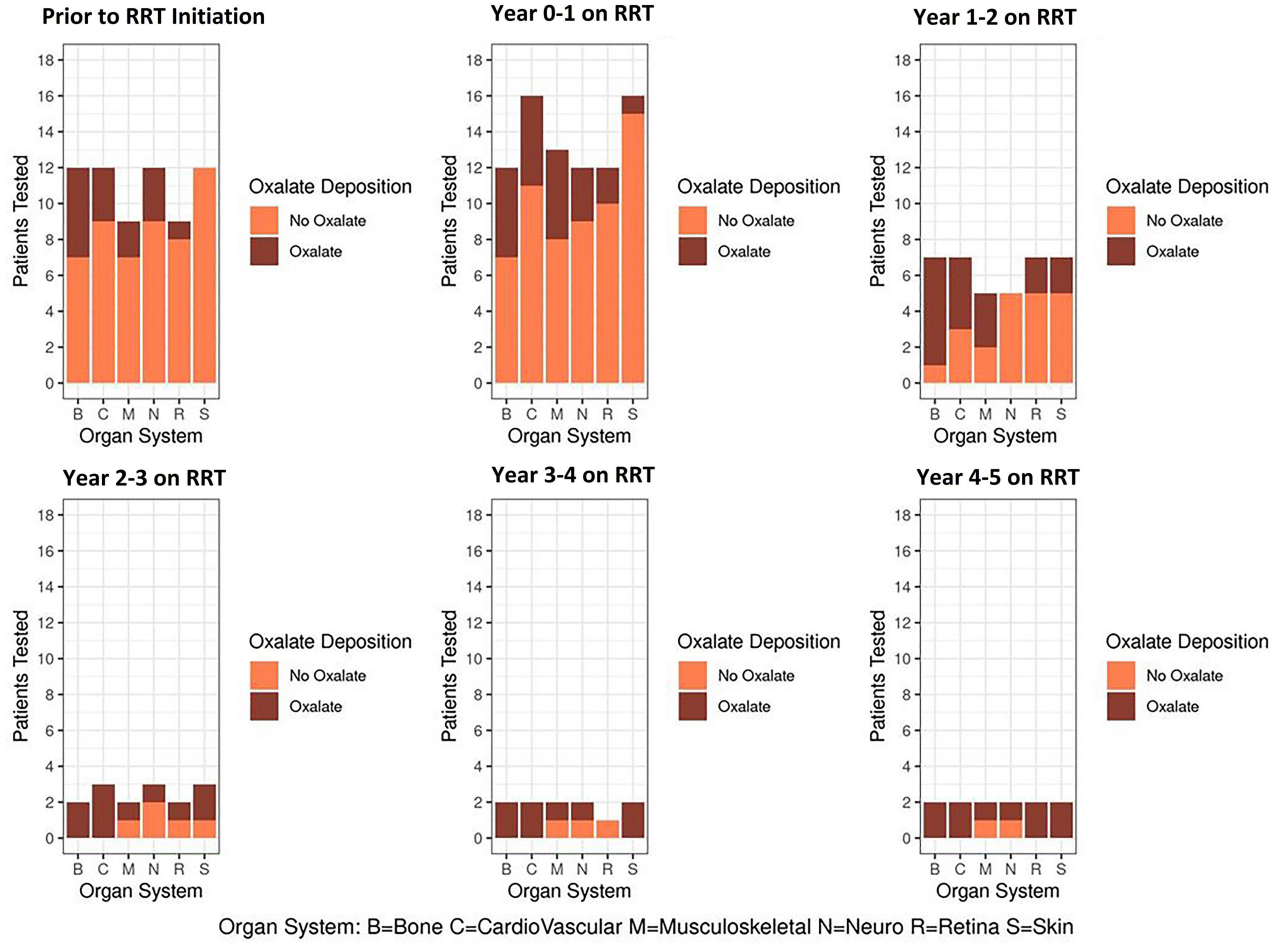
Clinical evidence of oxalosis over time on RRT.

As a group, PH1 patients had increasing symptoms or clinical testing evidence of progressive systemic oxalate deposition over time on RRT in all body systems including bone, cardiovascular, musculoskeletal, neurological, retina, and skin (Figure 4).

Among the 17 patients studied, 9 had no oxalosis-related symptoms. Three of these 9 also had no objective findings suggestive of systemic oxalosis on clinical, laboratory, nor imaging studies. An additional 5 had findings that were thought related to ESKD or other causes rather than to oxalate deposition (osteopenia alone, grade 1–2 diastolic dysfunction on echocardiography, advanced degenerative joint disease, mild sensory neuropathy). The remaining asymptomatic patient had retinal oxalate deposits without change in vision.

Among 7 patients who had symptoms and findings consistent with systemic oxalosis, severe hypotension complicating dialysis was observed in 3, overt cardiomyopathy or complete heart block in 4, marked musculoskeletal pain and weakness with or without neuropathy in 4, compromise of vision related to retinal oxalate deposits in 1, and complex metabolic bone disease with fractures in 2.

One remaining patient, an infant, had complex metabolic bone disease, growth, and developmental delay that were consistent with ESKD in this age group, though could have been complicated by systemic oxalate deposition.

Five of the patients underwent biopsies during the course of RRT, among whom 3 had calcium oxalate crystals confirmed by bone marrow biopsy. One of the 3 also had documented calcium oxalate crystals in a myocardial biopsy. The remaining biopsies of bone and a thyroid mass were negative for calcium oxalate crystals.

Of the 17 subjects in this cohort, 13 had echocardiograms during the RRT period (Table 4). Reference ranges for echocardiographic indices are found in Table 5 and descriptive statistics for the cohort are displayed in Table 6. There were a total of 30 echocardiography periods with at least partial data. The timing of the echocardiograms ranged from 0.6 to 33.6 months after the start of RRT [mean(SD) 11(8.6)], with time of first echocardiogram observation ranging from 0.6 to 12.5 months after RRT start and time to last echocardiogram observation 1.2–33.6 months after start of the RRT interval.

**Table 4:** Demographic data for PH1 patients on RRT who underwent echocardiography.

	**Overall (*N* = 13)**
**Age at RRT Start Date**	
Median (IQR)	30.7 (22.5, 66.0)
Range	18.5–75.9
**Age at Diagnosis**	
Median (IQR)	18.9 (5.0, 28.1)
Range	3.3–74.0
**Gender**	
Female	7 (53.8%)
Male	6 (46.2%)
**Race**	
White	12 (92.3%)
Unknown	1 (7.7%)
**Ethnicity**	
Non-Hispanic or Latino	7 (53.8%)
Unknown or not reported	6 (46.2%)
**Echocardiograms/subject**	
Median (IQR)	1.0 (1.0, 3.0)
Range	1.0–7.0
**Months to first echocardiogram**	
Median (IQR)	3.4 (2.8, 4.8)
Range	0.6–12.5
**Months to last echocardiogram**	
Median (IQR)	9.9 (2.9, 19.7)
Range	1.2–33.6

**Table 5:** Reference ranges for echocardiography measures.

**Echocardiogram parameter**	**Reference range**
Left ventricular ejection fraction	>50%
Left ventricular stroke volume	N/A – reference LVSVI
Left ventricular stroke volume index	>35 mL/m2
Left atrial volume index	<=34 mL/m2
Left ventricular mass index	Women 43–95 g/m2, men 49–115 g/m2
E/e'	Normal <10, 10–14 indeterminate, >14 abnormal
RV global strain	No lower limit to <-20
LV global strain	No lower limit to <-18
Right ventricular systolic pressure	>35 mmHg

*RV, Right Ventricular; LV, Left Ventricular; LVSVI, Left Ventricular Stroke Volume Index*.

**Table 6:** Descriptive echocardiographic data for PH1 patients on RRT.

	**Overall (*N* = 30)**
**Left ventricular ejection fraction**	
*N*	30
Median (IQR)	60.5 (56.0, 65.0)
Range	32.0–69.0
**LVEF in ref range**	
*N*	30
No	3 (10.0%)
Yes	27 (90.0%)
**Left ventricular stroke volume**	
*N*	28
Median (IQR)	87.0 (72.0, 99.5)
Range	63.0–152.0
**Left ventricular stroke volume index**	
*N*	28
Median (IQR)	43.0 (38.0, 51.5)
Range	33.0–72.0
**LVSVI in ref. range**	
*N*	28
No	2 (7.1%)
Yes	26 (92.9%)
**Left atrial volume index**	
*N*	26
Median (IQR)	33.5 (27.0, 43.2)
Range	16.0–64.0
**LAVI in ref. range**	
*N*	26
No	14 (53.8%)
Yes	12 (46.2%)
**Left ventricular mass index**	
*N*	29
Median (IQR)	94.5 (74.5, 112.0)
Range	55.0–169.0
**LVMI in ref. range**	
*N*	29
No	10 (34.5%)
Yes	19 (65.5%)
**E/e'**	
*N*	28
Median (IQR)	11.3 (8.0, 13.7)
Range	5.4–27.5
**E/e' interpretation**	
*N*	28
Normal	11 (39.3%)
Indeterminate	11 (39.3%)
Abnormal	6 (21.4%)
**RV global longitudinal strain**	
*N*	20
Median (IQR)	−24.5 (−28.0, −22.8)
Range	−32.0–−18.0
**RVGS in ref. range**	
*N*	20
**4 (20.0%)**	
Yes	16 (80.0%)
**LV global longitudinal strain**	
*N*	21
Median (IQR)	−19.0 (-20.0, −15.5)
Range	−22.0–−10.0
**LVGS in ref. range**	
*N*	21
No	9 (42.9%)
Yes	12 (57.1%)
**Right ventricular systolic pressure**	
*N*	28
Median (IQR)	34.0 (30.0, 39.0)
Range	18.0–73.0
**RVSP in ref. range**	
*N*	28
No	11 (39.3%)
Yes	17 (60.7%)
**Basal Mean**	
*N*	21
Median (IQR)	−16.3 (−19.5, −14.2)
Range	−22.8–−7.2
**BMS in ref. range**	
*N*	21
No	14 (66.7%)
Yes	7 (33.3%)
**Relative apical strain**	
*N*	21
Median (IQR)	0.6 (0.5, 0.7)
Range	0.3–0.9
**AS ratio <1**	
*N*	21
Yes	21 (100.0%)
**SBP**	
*N*	30
Median (IQR)	126.0 (110.0, 137.5)
Range	88.0–170.0
**DBP**	
N	30
Median (IQR)	71.0 (60.0, 78.0)
Range	52.0–92.0

*(N reflects echocardiography readings; some individual patients had multiple readings)*.

**Figure 5:**
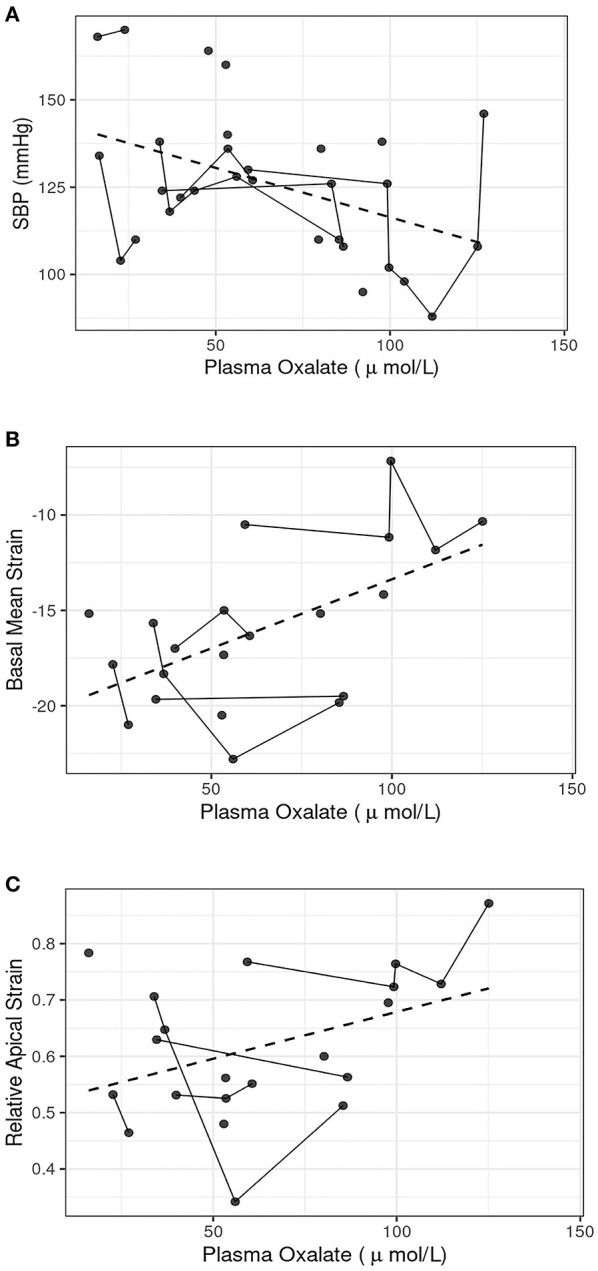
Regression models of selected markers of cardiac function as a function of pOx. Dashed line represents a simple linear regression fit. **(A)** Systolic blood pressure: Systolic blood pressure decreased at higher pOx, *p* < 0.05, *n* = 30 readings in 13 subjects. **(B)** Basal mean strain: Mean longitudinal strain of the basal segments of the LV plus RV worsened at higher pOx, *p* < 0.05, *n* = 21 readings in 10 subjects. **(C)** Relative apical strain: Trend toward apical sparing, *p* = 0.070, *n* = 21 readings in 10 subjects.

Regression model analysis results related to time on RRT and pOx are shown in Table 7. Only DBP showed a statistically significant change, with decline over time on RRT (estimate [95%CI] −0.54 [−0.97, −0.11], *p* < 0.05). Sensitivity analyses of subject-specific influence on model estimates indicated one subject contributed significant decreases in model estimates for E/e', LAVI, LVMI, and nearly significant for RVSP, and another subject contributed significant positive increases for LVEF, as well as large positive increases for LVMI and LVSVI. By contrast, regression models using pOx demonstrated decreasing systolic blood pressure as pOx increased (*p* < 0.05) (Figure 5A), lower LVSVI (*p* < 0.05), worsening of LV (*p* < 0.05), and RV global longitudinal strain (*p* < 0.05), as well as a trend toward lower DBP (*p* = 0.075). The mean longitudinal strain of the basal segments of the LV and RV worsened (*p* < 0.05) (Figure 5B) with a trend toward apical sparing (*p* = 0.07) (Figure 5C).

**Table 7:** Association of changes in echocardiography measures related to time and pOx on RRT in PH patients in linear regression models (up to 30 time points in 13 patients).

**CV parameter**	**Est(months on RRT)**	**CI**	**p-value**
RV global longitudinal strain	0.014	−0.192, 0.219	0.89
LV global longitudinal strain	−0.041	−0.187, 0.105	0.57
E/e'	0.118	−0.114, 0.35	0.31
LVEF	−0.252	−0.596, 0.092	0.14
LVMI	0.445	−0.842, 1.732	0.49
LVSVI	0.119	−0.423, 0.661	0.66
LAVI	0.303	−0.255, 0.861	0.27
RVSP	0.1	−0.379, 0.579	0.67
basal mean	−0.09	−0.291, 0.112	0.36
Apical mean	0.037	−0.125, 0.199	0.64
Relative apical strain	−0.002	−0.009, 0.004	0.44
SBP	−0.619	−1.551, 0.313	0.18
DBP	−0.539	−0.987, −0.092	0.020
**CV parameter**	**Est(pOx)**	**CI**	**p-value**
RV global longitudinal strain	0.061	0.012, 0.109	0.018
LV global longitudinal strain	0.046	0.006, 0.086	0.025
E/e'	−0.027	−0.089, 0.036	0.39
LVEF	−0.065	−0.155, 0.025	0.15
LVMI	−0.002	−0.337, 0.334	0.99
LVSVI	−0.184	−0.306, −0.061	0.005
LAVI	0.126	−0.033, 0.286	0.12
RVSP	−0.04	−0.172, 0.093	0.55
Basal mean	0.072	0.021, 0.124	0.008
Apical mean	0.029	−0.018, 0.076	0.21
Relative apical strain	0.002	0, 0.003	0.070
SBP	−0.283	−0.509, −0.056	0.016
DBP	−0.11	−0.232, 0.012	0.075

*RV, Right ventricular; LV, left ventricular; LVEF, left ventricular ejection fraction; LVMI, left ventricular mass index; LVSV, left ventricular stroke volume; LVSVI, left ventricular stroke volume index; LAVI, left atrial volume index; RVSP, right ventricular systolic pressure; SBP, Systolic Blood Pressure; DBP, Diastolic Blood Pressure*.

Echocardiography was performed in two additional pediatric patients according to a pediatric echocardiography protocol. Values were not included in the averages because of the young ages of these patients and age-related differences in echocardiographic measurements. One patient, age 11 months, had left ventricular ejection fraction of 65% and mild-moderate concentric left ventricular hypertrophy. There was no evidence of coarctation of the aorta. Medial e' was low at 0.05 m/s and E/e' was indeterminate at 12. Strain was not measured. The other patient, age 2 years, had left ventricular ejection fraction ranging between 64 and 69% on two serial echocardiograms. Medial e' was normal at 0.10 and E/e' was normal at 6. Left ventricular GLS was attempted on the second echocardiogram and an average value of the 12 visualized segments was normal at −22.

## Discussion

Supersaturation of calcium oxalate in blood leading to systemic oxalosis is thought to occur when pOx increases to >30–45 μmol/L (3, 4). Our data show that most patients with PH1 maintain pOx concentrations above this limit of supersaturation with no clinically significant decline over time despite intensified RRT. Standard hemodialysis prescriptions likely lead to unacceptably high pOx and high risk for worsening systemic oxalosis. This observation alone highlights the challenge for both clinicians and patients in managing PH1, the need for thoughtful, individualized management including regular pOx measurement to determine the efficacy of the dialysis prescription, and the urgent need for more efficacious therapy. Our data also show that PH1 patients are at risk for developing cardiac dysfunction while on RRT, illustrating one potential consequence from systemic oxalosis.

Previous work from the Rare Kidney Stone Consortium and other investigators has shown that, even though HD effectively clears oxalate from plasma during a given treatment, pre-dialysis pOx concentrations are variable and often remain quite elevated due to oxalate re-entering circulation from other compartments (3, 8, 9). Our study expands upon these observations by providing an analysis of serial pOx values obtained from a relatively large cohort of PH1 patients maintained on RRT for 1–5 years. Our previous work illustrated the importance of maintenance of residual urine output given the decline in oxalate excretion as urine output diminishes, and data from this study are consistent with this, though our sample size is too small to generate statistical significance (8).

When treating patients with PH1 and ESKD, the clinician is challenged with balancing the risks of systemic oxalosis with the burden more frequent and longer dialysis sessions impose on a patient. While nocturnal home HD might be another option to manage ESKD (10), it has not been rigorously studied and the efficacy might depend largely upon the dialysis system available for use since volume flows of dialysate vary in home systems (11). The risks of elevated pOx and prolonged time to transplant from starts of RRT are associated with an increased risk of post-transplant complications in PH, particularly in regards to rapid recurrence of oxalosis in the transplanted kidney (12).

The current study demonstrates that clinically evident manifestations of oxalate on bone, skin, nervous system, and eyes vary from patient to patient over time on dialysis (13–18). Due to our small sample size and retrospective review of clinically-indicated testing for oxalosis, we cannot draw statistically significant conclusions regarding prevalence. However, our data suggest that this patient population is susceptible to multi-tissue involvement from oxalate deposition and that the number of organ systems per subject increases over time on dialysis. The dynamic equilibrium between oxalate in plasma and deposits of oxalate in tissue is not well-understood; it is possible that accumulating oxalate tissue deposits attenuate the amount of oxalate circulating in plasma.

Most symptoms as well as laboratory and imaging findings of systemic oxalosis can mimic those of ESKD. For example, complex metabolic bone disease and cardiac dysfunction are characteristic of all patients maintained on chronic RRT. Tissue biopsies are not often performed, thus indirect methods that lack specificity are usually relied upon for oxalosis detection. Thus, our retrospective study may overestimate the frequency of certain aspects of oxalosis.

Cardiac manifestations in PH1 patients are described in the literature, though most are anecdotal observations. Mookadam et al. found that 82% of PH patients had cardiac abnormalities by either echocardiography or electrocardiography, with increased LVMI and left atrial enlargement being most common (19). Conduction disturbances including bundle branch block and atrioventricular block were also observed in that study. Quan and Biblo described a PH patient with ventricular tachycardia and valvular dysfunction (20). In 2013, Lagies et al. described a PH1 patient with apical sparing of longitudinal strains, left ventricular rotational abnormalities, and short-axis dysfunction along with characteristics of infiltrative cardiomyopathy with restrictive physiology (21). A more recent publication from the same group reports impaired global longitudinal strain despite preserved left ventricular ejection fraction in 15 PH patients (13 PH1, 1 PH2, and 1 PH3) not on RRT, demonstrating subclinical myocardial disease and supporting early monitoring of cardiac function in PH patients (22).

Intracardiac deposition of calcium oxalate crystals would be expected to result in progressive worsening of cardiac function. Mode sensitive markers of ventricular function, namely left ventricular and right ventricular global longitudinal strain did not worsen over the course of RRT in the group as a whole. However, worse global longitudinal strain was associated with higher plasma oxalate. This is of particular concern given that abnormal global longitudinal strain leads to increased risk for cardiovascular morbidity and mortality (23, 24). Basal strain showed particular worsening, with a trend toward apical sparing as pOx increased in our patients. Thus, the functional consequences of calcium oxalate deposition in cardiac tissue appear similar to that of infiltration with amyloid proteins. In cardiac amyloidosis, longitudinal strain in the basal ventricular segments typically deteriorates first; apical segments may be spared despite the systemic nature of the disease (25, 26). We also observed a decline in blood pressure over the course of RRT overall, with 3 patients developing severe hypotension that complicated dialysis. Systolic blood pressure correlated inversely with pOx with a similar trend for diastolic pressure.

Published reports of echocardiography in non-PH patients maintained on HD describe some degree of myocardial impairment despite preserved ejection fraction (27–29). Lagies et al. also reported abnormal longitudinal cardiac rotation and left ventricular longitudinal strain in a significant proportion of a cohort of HD patients (30). It is possible that oxalosis may impact myocardial function differently from general ESKD and RRT. Further investigation regarding impaired myocardial function in PH patients on HD compared to non-PH patients on HD is warranted to further delineate the impact of PH on cardiac function compared to HD alone. Our findings showing worsening diastolic function and worsening of left ventricular global longitudinal strain correlating with pOx suggest the importance of serial echography for this patient population.

Our data demonstrating a sustained elevation in pOx and progressive systemic oxalate deposition over the duration of intensive RRT highlight the urgency for new innovative therapies for PH. Standard RRT alone is not effective for many PH patients to achieve an acceptable pOx; thus, treatment options that reduce oxalate production or enhance removal would greatly benefit this patient population. Moreover, data show that after PH1 patients maintained on HD receive a liver and kidney transplant, urinary oxalate excretion remains quite high for a long period of time (31), posing potential risk to the transplanted allograft, providing further evidence that strategies to more effectively lower pOx while maintained on HD are needed.

Urine volume and oxalate excretion were maintained in a subset of our PH1 patients despite markedly reduced GFR. Thus, renal excretion provides another critical opportunity for additional elimination of oxalate. Strategies to maintain urine volume, including avoidance of aggressive fluid removal during dialysis sessions and generous oral intake between dialysis sessions should be considered where appropriate.

Our study has some limitations. Most importantly, the retrospective nature limited the number and timing of clinical variables and tests that were available for analysis. These patients also were seen at a single tertiary medical center though ongoing care was provided by local dialysis centers, so the results and frequency of outcomes might not transfer to all settings. In addition, there may have been selection bias regarding who received which tests over time based upon the clinical situation. Our data set lacks objective measures of dialysis delivery like Kt/V, so we relied on hours of dialysis, acknowledging that it would have been more meaningful to show suboptimal pOx in the context of proven adequate Kt/V. Also, our echocardiography cohort had a wide range of ages, so we cannot exclude that some of our findings may be related to age differences. Nevertheless, this is one of the largest PH1 cohorts reported to date maintained on HD for a prolonged period of time.

## Conclusions

Patients with PH1 are exposed to persistently high pOx concentrations even when they receive an intensified dialysis regimen. Thus, they are increasingly at risk for systemic oxalosis in multiple organ systems over time on RRT. Failure to recognize the risks of insufficient dialysis in PH1 patients may have significant consequences. Echocardiographic data suggests worsening of diastolic dysfunction over time. Decline in blood pressure and worsening of basal ventricular strain consistent with an ongoing infiltrative process correlated with higher pOx. These observations support an urgent need for improved management strategies for PH1 patients who develop ESKD.

## Data Availability Statement

The raw data supporting the conclusions of this article will be made available by the authors, without undue reservation.

## Ethics Statement

The studies involving human participants were reviewed and approved by Mayo Clinic Institutional Review Board. Written informed consent to participate in this study was provided by the participants' legal guardian/next of kin.

## Author Contributions

DS, JL, DM, and FE: contributed to the research idea, study design, data analysis, interpretation, and manuscript preparation. FE, TG, and RM contributed to data analysis, interpretation, and performed statistical analysis. JO, BS, and CB contributed data acquisition and subject recruitment. BD provided research idea, data analysis, interpretation, and content feedback. PP contributed expertise in cardiology content and data analysis, interpretation. Each author contributed important intellectual content during manuscript drafting or revision and accepts accountability for the overall work by ensuring that questions pertaining to the accuracy or integrity of any portion of the work are appropriately investigated and resolved.

## Conflict of Interest

BD is an employee of OxThera. The remaining authors declare that the research was conducted in the absence of any commercial or financial relationships that could be construed as a potential conflict of interest.
